# Assessment of the Surface Characteristics of ISO 5832-1 Stainless Steel for Biomaterial Applications

**DOI:** 10.3390/ma18174020

**Published:** 2025-08-27

**Authors:** Eurico Felix Pieretti, Davide Piaggio, Isolda Costa

**Affiliations:** 1 Nuclear and Energy Research Institute, São Paulo 05508-000, Brazil; 2 School of Engineering, University of Warwick, Coventry CV4 7AL, UK

**Keywords:** bioengineering, cytotoxicity, marking process, Nd:YAG laser, tribology

## Abstract

Marking techniques are employed to guarantee the identification and traceability of biomedical materials. This study investigated the impact of laser and mechanical marking processes on the tribological performance of ISO 5832-1 austenitic stainless steel (SS), specifically examining corrosion resistance, the coefficient of friction, and wear volume in ball-cratering wear tests. The laser marking was performed using a nanosecond Q-switched Nd:YAG laser. Cytotoxicity tests assessed the biocompatibility of the biomaterial. Non-marked surfaces were also evaluated for comparison. A phosphate-buffered saline solution (PBS) served as both the lubricant and corrosion medium. The surface finishing was analyzed using optical microscopy and scanning electron microscopy coupled with a field-emission gun (SEM-FEG), combined with an energy-dispersive X-ray spectrometer. The oxide film was examined through X-ray photoelectron spectroscopy (XPS). Wear tests lasted 10 min, with PBS drops applied every 10 s at 75 rpm; solid balls of AISI 316L stainless steel (SS) and polypropylene (PP), each 1 inch in diameter, were used as counter-bodies. Corrosion resistance was assessed using electrochemical methods. Results showed variations in roughness and microstructure due to laser marking. The tribological behaviour was influenced by the type of marking process, and the wear amount depended on the normal force and ball nature. None of the samples was considered cytotoxic, although laser-marked surfaces exhibited the lowest cellular viability among the tested surfaces and the lowest corrosion resistance.

## 1. Introduction

Implantable medical devices for joints and dental use must be biocompatible, strong, and resistant to corrosion. Bodily fluids subject these implants to harsh conditions and variable loads, leading to possible particle release from corrosion, fatigue, or friction, which can cause patient complications if particles migrate through body fluids. Okazaki [1] studied how friction affects the anodic polarization of metallic biomaterials.

Metallic particles produced during corrosion can migrate through the circulatory system or tissue, either passively or via active transport [2], which may impact biocompatibility. Anderson [3] observes that a biomaterial’s biocompatibility may vary depending on its application.

Several characteristics influence how metal implants affect the human body, including metal surface structure, mechanical properties, size, and shape. Key factors include wear particles in the body, hydration levels, physical stress on both the body and implant, and issues like surface corrosion and oxidation [3], which are relevant to this work.

Orthopedic implants are engineered and produced to ensure that, when utilized as intended and under appropriate conditions, any associated risks are considered acceptable relative to the anticipated benefits afforded to patients.

ISO 5832-1 stainless steel is extensively used in implant manufacturing due to its good mechanical, metallurgical, and electrochemical properties, as well as its affordability [4,5]. Stainless-steel biomaterials serve as either permanent or temporary implants to support bone healing. Marking techniques are employed for identification or traceability purposes [4,5,6,7]. Laser and mechanical engravings are preferred methods due to their automation, ease of use, reproducibility, and low cost [4,5]. Laser marking reduced the biomedical-grade stainless steel’s resistance to localized corrosion, making it more susceptible to pitting compared to mechanical methods. This effect is associated with alterations in passive oxide properties, surface morphology, and chemical composition [4,5,6].

The ball-cratering scratch test (or micro-scale wear test) is an effective approach for analyzing the tribological resistance of many materials and alloys [8,9,10]. The ball-cratering scratch test has been extensively utilized in studies examining the abrasive wear behaviour of different materials [11,12,13,14,15].

Wear trials conducted using the ball-cratering procedure offer improvements over other techniques, as they can be carried on with normal forces (N) and ball rotations (n) that are fairly low (N < 0.5 N and n < 80 rpm) [16,17,18,19,20,21].

Topographic features directly affect various surface properties, including electrochemical and tribological behaviours. Rougher surfaces are more prone to localized corrosion and have lower mechanical strength [22,23]. Corrosion is the process by which metallic materials degrade due to their chemical reactions with the environment [24,25]. Some metals, such as iron, titanium, aluminum, copper, chromium, and nickel, develop a passive film on their surface that can slow corrosion through a barrier effect and cathodic protection [26].

This study aims to assess the cytotoxicity, corrosion resistance, and tribological behaviour of the ISO 5832-1 austenitic stainless steel (SS) marked using laser and mechanical techniques through a ball-cratering wear method and electrochemical tests.

## 2. Experimental

### 2.1. Materials

Specimens of ISO 5832-1 austenitic stainless steel (chemical composition (wt%): 0.023 C, 0.78 Si, 0.0003 S, 2.09 Mn, 0.026 P, 14.33 Ni, 18.32 Cr, 2.59 Mo, and Fe balance) were marked mechanically and with a Q-switched nanosecond pulsed Nd:YAG laser at a wavelength of 1064 nm, with a scanning speed of 4 mm s^−1^ and a frequency of 20 Hz. Balls made of AISI 316L stainless steel and polypropylene (PP) with a diameter of D = 25.4 mm (D = 1”) were used.

A PBS (phosphate-buffered saline) solution with a chemical composition (g/L) of 0.2 KCl, 8.0 NaCl, 0.2 KH_2_PO_4_, and 1.15 Na_2_HPO_4_, a conductivity of 15.35 mS, and a pH value of 7.4 was applied as a lubricant between the specimen and the ball. Table 1 presents the hardness (H) of the biomaterials used in this study (balls and specimens).

### 2.2. Ball-Cratering Wear Test Equipment and Data Acquisition

An apparatus with a free-ball pattern was used for the sliding tribological tests. Two load cells were employed in the ball-cratering apparatus, with one to measure the tangential force (T) generated during the experiments and another to control the normal force (N). The “tangential” force and the “normal” load cells have a maximum capacity of 50 N and an accuracy of 0.001 N. The values of “N” and “T” are read by a readout system.

**Figure 1:**
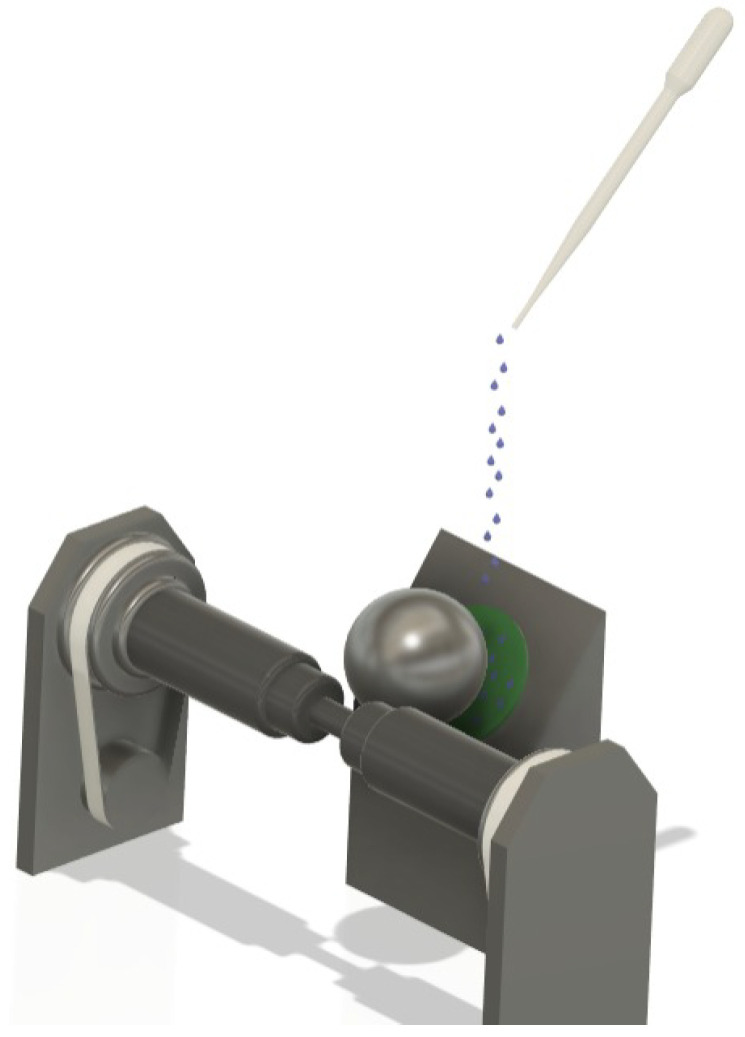
Three-dimensional schematic representation of the operational principle of the ball-cratering tribological test.

Figure 1 illustrates a schematic diagram of the wear test principle, where a rotating ball is pressed against the specimen being tested. Simultaneously, a lubricant is supplied between the ball and the specimen during the experiments. The ball-cratering wear test aims to create “wear craters” on the specimen. The wear volume (V) can be calculated based on the wear crater radius (b) using Equation (1) [11], where R is the ball’s radius. Table 2 summarizes the experimental test conditions.

(1) $$V≅\frac{\pi b^{4}}{64R}\;\mathrm{for}\;b<<R$$

Based on the density (*ρ*) of the material of the ball (AISI 316L SS: *ρ*_316L_ = 8 g/cm^3^; polypropylene: *ρ*_PP_ = 0.91 g/cm^3^), the values of the normal force (*N*) for the wear experiments were defined as *N*_316L_ = 0.40 N and *N*_PP_ = 0.05 N.

The rotational speed of the ball was n = 75 rpm. For n = 75 rpm and D = 25.4 mm (R = 12.7 mm), the tangential sliding speed at the ball’s external diameter was equal to v = 0.1 m/s.

The tribological tests were conducted during t = 10 min, with the values of v = 0.1 m/s and t = 10 min (t = 600 s), which calculated a sliding distance of S = 60 m.

The tests were performed without interruption, with the chemical solution agitated and supplied between the specimen and the ball at a rate of one drop every ten seconds throughout the process.

The normal force (N) was applied to the specimen using a servo-motor/servo-controller and measured by a load cell. The tangential force (T), obtained during testing, was recorded by a second load cell beneath the specimen and displayed on a readout system. The coefficient of friction (COF) was calculated using Equation (2) [11].

(2) $$\mu =\frac{T}{N}$$

All tribological experiments were performed three times per sample to confirm the reproducibility of the results (n = 3). Data were assessed using a two-way ANOVA followed by a Tukey test. (n = 3). (*p* < 0.05).

### 2.3. Surface Characterizations

Surface studies were performed using an optical microscope (Olympus, TM, Olympus Corporation, Shibuya, Tokyo, Japan) and a scanning electron microscope paired with a high-resolution field emission gun (SEM-FEG, FEI—INSPECT F50, Thermo Fisher Scientific, Hillsboro, OR, USA). The chemical composition was determined through energy-dispersive X-ray (EDX) semi-quantitative analysis and X-ray photoelectron spectroscopy (XPS), obtained using a Kratos XSAM HS (Kratos Analytical, Manchester, UK) equipment under an ultra-high vacuum (*p* ≈ 3 × 10^−8^ Torr). The excitation source was magnesium K, with an energy of 1.2536 keV [27].

### 2.4. Cytotoxicity Analysis

Cytotoxicity was evaluated using a quantitative method. The assay involves measuring cell viability after exposing the cell population to various concentrations of the extract, which was obtained by incubating the specimens in RPMI (Gibco^®^, Thermo Fisher Scientific, Hillsboro, OR, USA) cell culture medium at 37 °C for 24 h under continuous stirring.

The analysis of viable cell numbers was conducted using a colorimetric method that involved the incorporation of supravital dye along with the electron-coupling agent (MTS/PMS), followed by spectrophotometric measurement at 490 nm. The amount of dye uptake by the cell population directly reflects the cell viability in culture.

The relationship between the concentration of the extract and the number of viable cells resulted in a dose–response curve, and the parameter used to assess cytotoxicity is calculated by the extract concentration that killed 50% of the exposed cells in the test (inhibitory concentration—IC50%).

The identification of the test substances was taken from ISO 5832-1 austenitic stainless steel with mechanical and laser marks; their controls included a “Positive Control: Phenol 0.5%” and a “Negative control: commercially pure titanium”. The system test used Chinese hamster ovary cells (CHO-K1 cell line), which are of epithelial type.

The preparation of extracts was according to ISO 10993-12. Serial dilutions with five concentrations were defined, namely 100%, 50%, 25%, 12.5%, and 6.25%. The net extractor was RPMI culture medium (Gibco^®^) with serum, and the incubation condition was 24 h at 37 °C with constant stirring. The test was conducted in quadruplicate in incubated plates for 24 h in a CO_2_ glasshouse at 37 °C.

The vital dye MTS/PMS was applied under the following conditions:

1. Solution of MTS/PMS prepared with PBS at a concentration of 20:1;
2. Solution of MTS/PMS culture medium with serum: 20 μL was added to the solution of MTS/PMS, and an additional 80 μL of culture medium was added to each test well.

Finally, the incubation was conducted in a glasshouse with CO_2_ for 2 h at 37 °C, and the reading was carried out in a microplate reader at 490 nm.

### 2.5. Electrochemical Experiments

Electrochemical measurements were conducted utilizing a potentiostat/galvanostat (PCI4/300, Gamry Instruments, Warminster, PA, USA). A standard three-electrode cell setup was used: the stainless-steel specimen served as the working electrode, a platinum thin wire as the counter-electrode, and the Ag/AgCl (KCl, 3 M) electrode as the reference electrode. The electrolyte was a phosphate-buffered saline solution (PBS) at 37 °C with pH 7.4. The open-circuit potential was monitored over time for 17 h (61200 s), after which the cyclic potentiodynamic polarization measurement was conducted at a scan rate of 0.167 mV·s^−1^. All electrochemical tests were repeated six times to ensure reproducibility (n = 6).

## 3. Results and Discussion

### 3.1. Wear Volume

Human joints are mainly lubricated through hydration and superlubrication processes, where water interacts with charged molecules to reduce friction. This involves forming hydration layers around charged molecules and surfaces within the joint. These water layers are attracted to charged surfaces, creating a film that enables low-friction movement. Hydration layers can withstand high pressures without being displaced. In joint lubrication, superlubrication refers to the extremely low friction levels observed in natural joints [28,29,30,31].

Natural joints, such as the knee and hip, are lubricated by synovial fluid, which contains hyaluronic acid and other molecules that contribute to lubrication through hydration. These molecules help maintain a low-friction environment, enabling the joint to move smoothly. Understanding the mechanisms of joint lubrication is crucial for developing artificial joints [28,29,30,31]. In this study, phosphate-buffered saline (PBS) solution served as both the wear lubricant and the electrolyte during corrosion testing.

**Figure 2:**
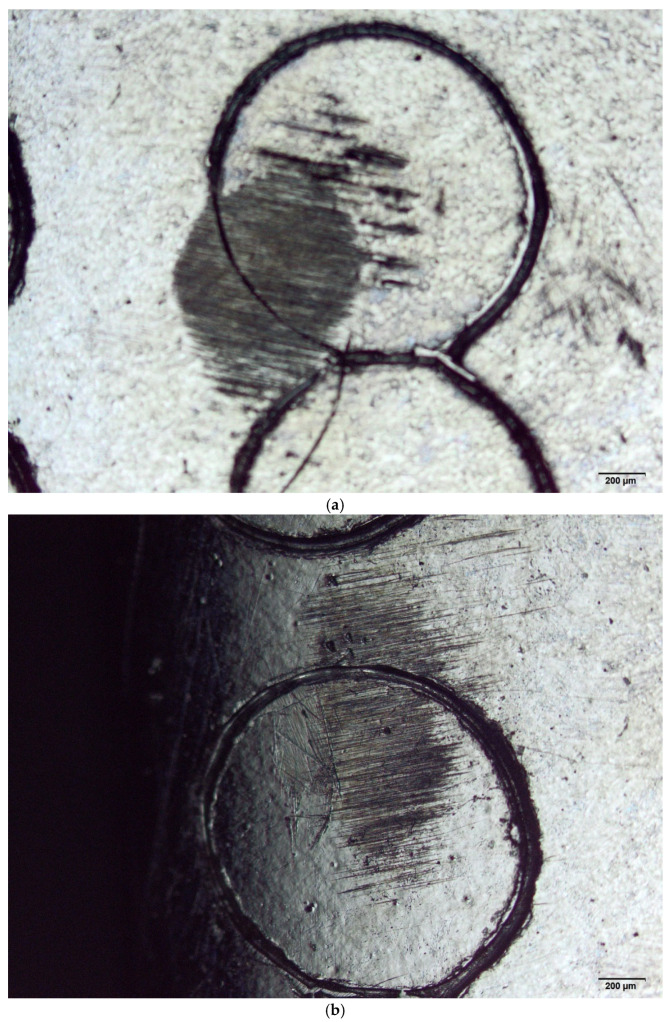

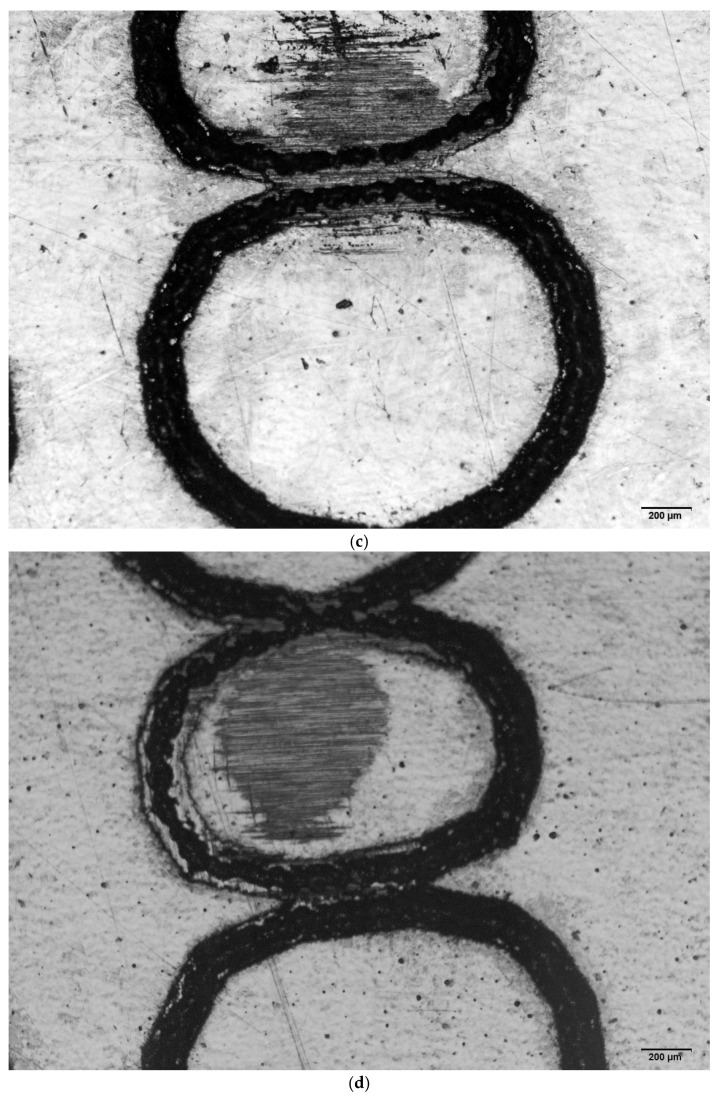

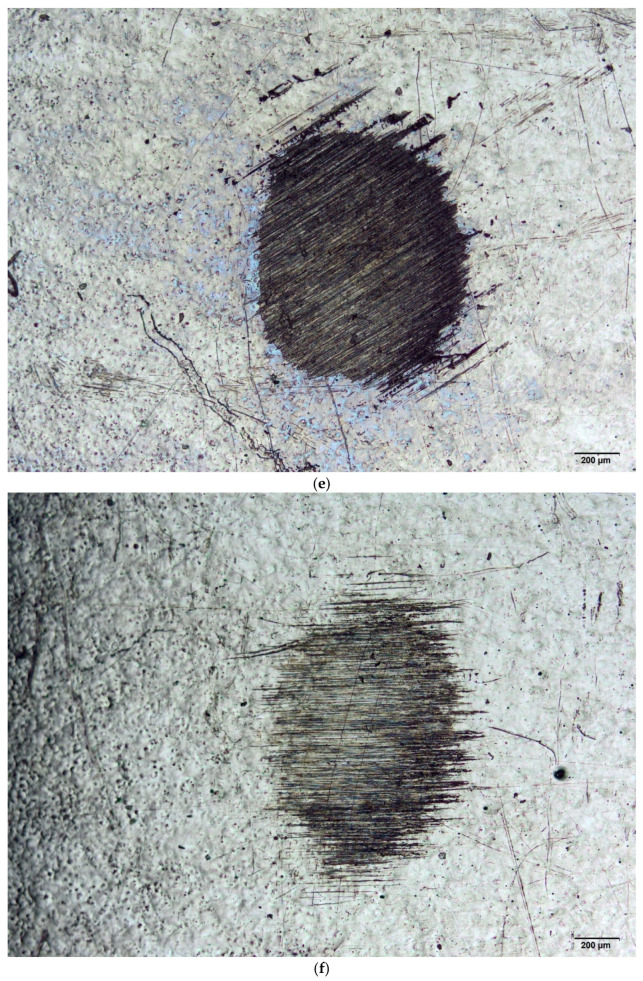
(**a**) Optical microscopy image showing the mechanically marked ISO 5832-1 SS sample after the wear test conducted with the AISI 316L SS ball with PBS lubricant. (**b**) Optical microscopy image showing the mechanically marked ISO 5832-1 SS sample after the wear test conducted with the polypropylene ball with PBS lubricant. (**c**) Optical microscopy image showing the laser-marked ISO 5832-1 SS sample after the wear test conducted with the AISI 316L SS ball with PBS lubricant. (**d**) Optical microscopy image showing the laser-marked ISO 5832-1 SS sample after the wear test conducted with the polypropylene ball with PBS lubricant. (**e**) Optical microscopy image presenting the ISO 5832-1 SS sample, without marks, after the wear test conducted with the AISI 316L SS ball with PBS lubricant. (**f**) Optical microscopy image presenting the ISO 5832-1 SS sample, without marks, after the wear test conducted with the polypropylene ball with PBS lubricant.

Figure 2a–f displays images of the specimens subjected to wear tests. Surfaces marked using laser and mechanical techniques were examined with optical microscopy. For comparison, the blank surfaces were also evaluated. These findings are important to the biomaterials field, as they assist in selecting appropriate areas for markings to prevent wear.

The significant disparity in the hardness of metallic and polymeric spheres is reflected in their respective normal force values. This comparison is relevant because implantable medical devices designed to replace human joints frequently utilize combinations of dissimilar materials, such as metal–polymer or metal–ceramic interfaces [1,2,3]. Each tribological test was performed three times per sample for reproducibility (n = 3).

**Figure 3:**
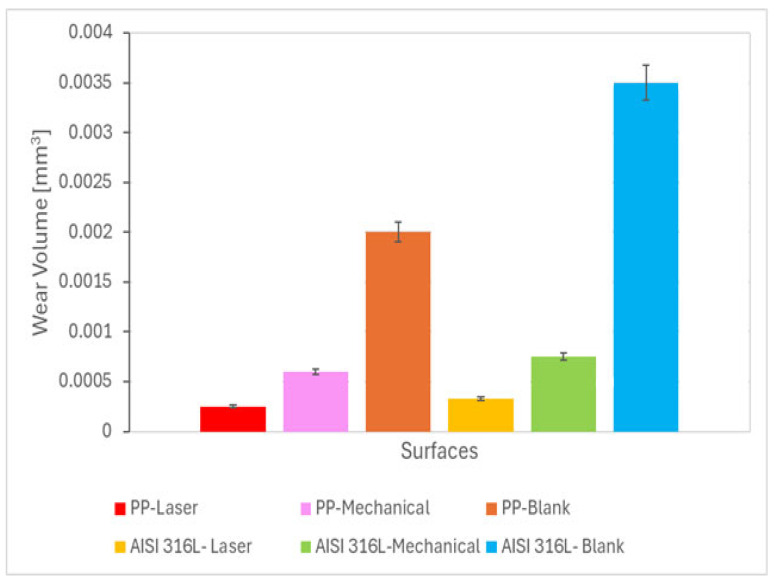
Wear volume as a function of the ball material and the type of marking process. Data were assessed using a two-way ANOVA followed by a Tukey test. (n = 3). (*p* < 0.05).

Figure 3 shows the wear volume (V) for the pristine and mechanically and laser-marked specimens. A decrease in the wear volume is demonstrated on the marked specimens. This decrease is associated with a possible increase in the hardness of the specimen due to localized heat treatment produced by the laser beam or to the mechanical work by the stamp.

Laser surface hardening is a procedure that utilizes a high-power laser beam to heat the surface of stainless steel rapidly. This rapid heating, followed by quenching, results in a phase transformation in the material’s microstructure, typically converting austenite to martensite, which has greater hardness and wear resistance. A focused laser beam allows for accurate control of the area being hardened, making it possible to modify specific locations without altering the overall properties of the material. The treated surface layer demonstrates increased hardness and wear resistance compared to the untreated material, leading to reduced wear volume loss in situations involving sliding or abrasive contact [32].

### 3.2. Analysis of Coefficient of Friction

**Figure 4:**
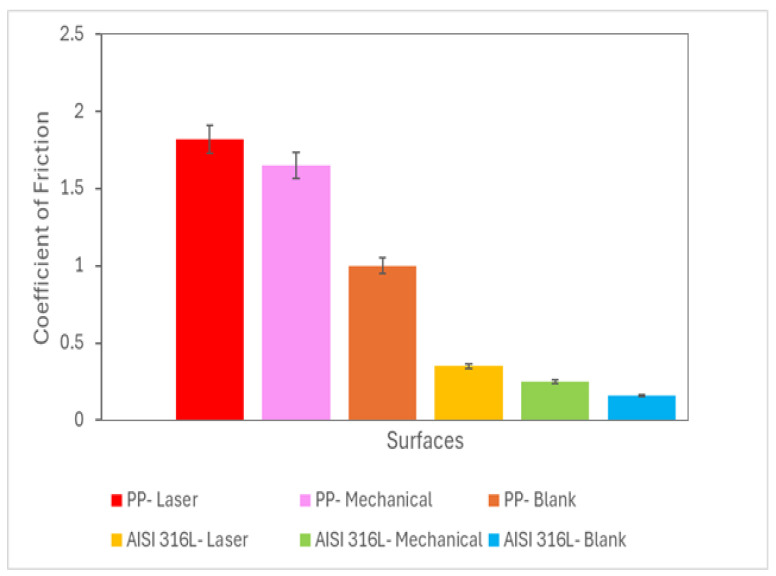
Coefficient of friction as a function of the ball material and the type of marking process. Data were assessed using a two-way ANOVA followed by a Tukey test. (n = 3). (*p* < 0.05).

Figure 4 shows the coefficient of friction (μ) values for the mechanical- or laser-marked specimens. The AISI 316L stainless-steel ball had a lower coefficient of friction than the polypropylene ball.

An increase in normal force resulted in a corresponding increase in wear. This behaviour was reported in the literature [33,34,35,36]. The highest wear volume values were reported for blank samples. Samples with marks made by mechanical methods or laser exhibited increased wear resistance.

Regarding the coefficient of friction, the highest values were observed using the polypropylene ball. Magnitudes of these values were reported elsewhere [12] with the same type of assay under another tribological system. The reduction in the coefficient of friction in the austenitic stainless-steel balls is due to the higher hardness of steel compared to polypropylene. The blank samples exhibited the lowest values, which is attributed to the smoothness of their surface. The coefficient of friction quantifies the resistance to sliding between two surfaces. A lower COF indicates better lubrication. A phosphate-buffered saline (PBS) solution, often used as a lubricant, can exhibit a coefficient of friction of approximately 0.1, which must be considered relatively high and can affect the wear response [31].

No correlation was observed between wear volume and the coefficient of friction; higher wear did not correspond to a higher coefficient of friction.

### 3.3. Cytotoxicity Analysis

**Figure 5:**
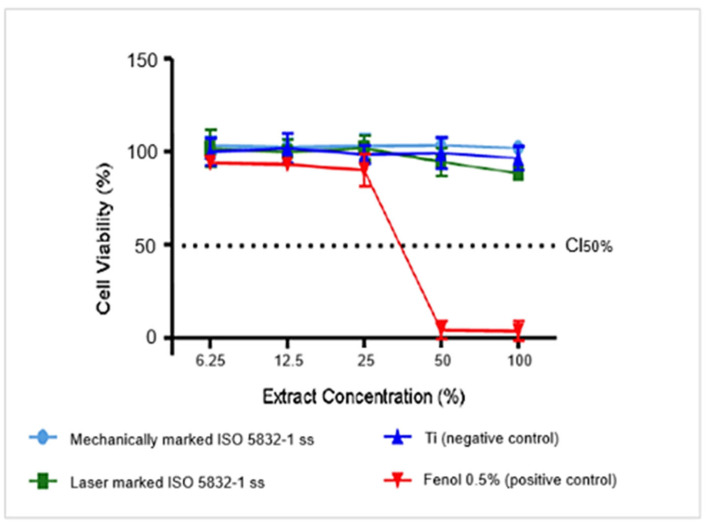
Cell viability vs. the extract concentration for samples engraved via laser and mechanical techniques.

Table 3 presents the pH values of the extracts measured at a concentration of 100%. The results presented in Figure 5 suggest that the ISO 5832-1 stainless-steel samples exhibited a similar behaviour to the negative control, meaning there was no cytotoxicity. The cell viability curves for both mechanically and laser-marked samples consistently remain above the IC50% cytotoxicity threshold. Blank specimens were not evaluated, as this alloy is commonly used for biomedical applications.

The cytotoxicity of ISO 5832-1 stainless steel was assessed in accordance with ISO 10993-5 [37]. Stainless steels intended for medical and dental applications must exhibit no cytotoxicity [38]. A slight decrease in cell viability was observed for the laser-marked specimens, which may be associated with their susceptibility to localized corrosion [4,5]. Since this method examines only surface characteristics and corrosion is a surface phenomenon, the laser marking procedure affects the susceptibility to corrosion of austenitic stainless steel, producing a less protective passive film with areas prone to its breakdown [4], although in this case, the specimens were considered non-cytotoxic, as they remained above the CI50% threshold for cytotoxicity.

### 3.4. Electrochemical Tests

Electrochemical measurements are essential for estimating the corrosion susceptibility of biomaterials with various surface finishes. The passive film formed on ISO 5832-1 stainless steel significantly enhances corrosion resistance. When the alloy contacts simulated body fluid, a less defective and more uniform passive film develops [39,40].

**Figure 6:**
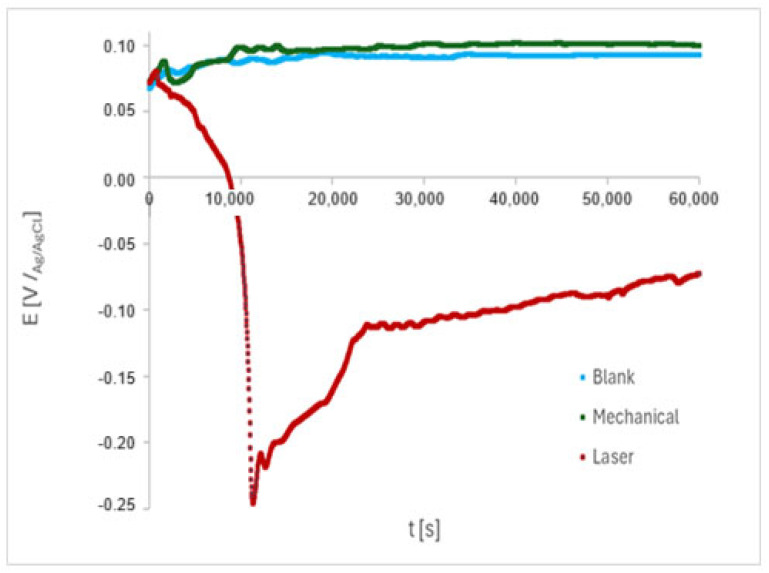
Variation in the OCP with the immersion time in PBS for the ISO 5832-1 stainless steel, with mechanically, laser-marked, and pristine samples. Total time: 61,200 s. (n = 6).

Initially, the open-circuit potential (OCP) was verified in PBS during 61,200 s, as shown in Figure 6. The Eocp shifted positively, indicating that a protective oxide layer begins to form on blank and mechanically marked samples, while it dropped to negative values on laser-marked samples during the initial immersion period. Subsequently, Eocp shifted to around −0.1 V/_Ag/AgCl_ and showed an increasing trend throughout the test for the laser-treated sample. For the blank and mechanically marked samples, Eocp was around 0.07 V/_Ag/AgCl_ at the start of the monitoring period, then shifted to nobler potentials, exhibiting a stabilizing trend throughout the test starting at approximately 30,000 s.

The OCP fluctuations are explained by the immediate competition between oxide layer formation and dissolution [41] during the initial immersion period. The passive film in laser-marked areas is uneven, and the appearance of micro-pits causes oscillations in the potential, which may be linked to micron defects formed during the laser melting process. Conversely, broken passive films are quickly re-passivated, leading to an increase in potential.

**Figure 7:**
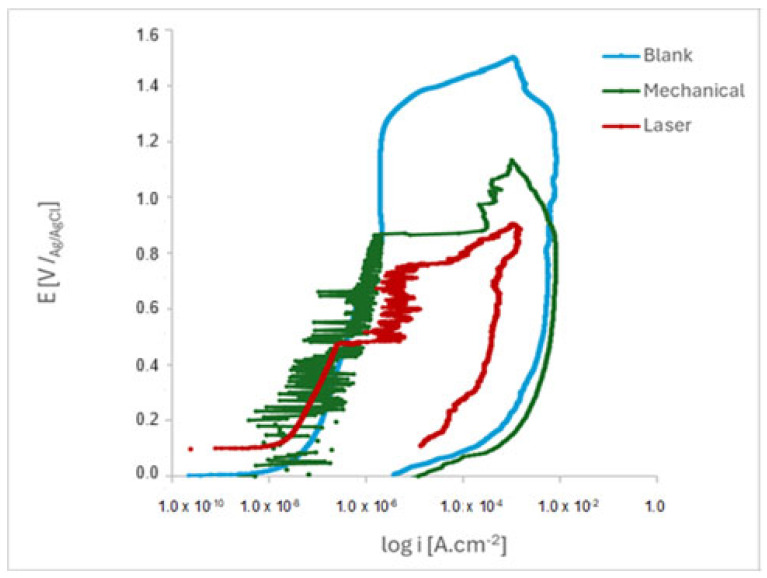
Cyclic potentiodynamic polarization curves of the ISO 5832-1 stainless-steel samples after 17 h of immersion in PBS (n = 6).

Figure 7 shows the anodic polarization curves for the marked and pristine ISO 5832-1 SS samples after 17 h of immersion in PBS. A low corrosion current density (icorr) corresponds to a reduced corrosion rate or increased corrosion resistance [42,43].

**Figure 8:**
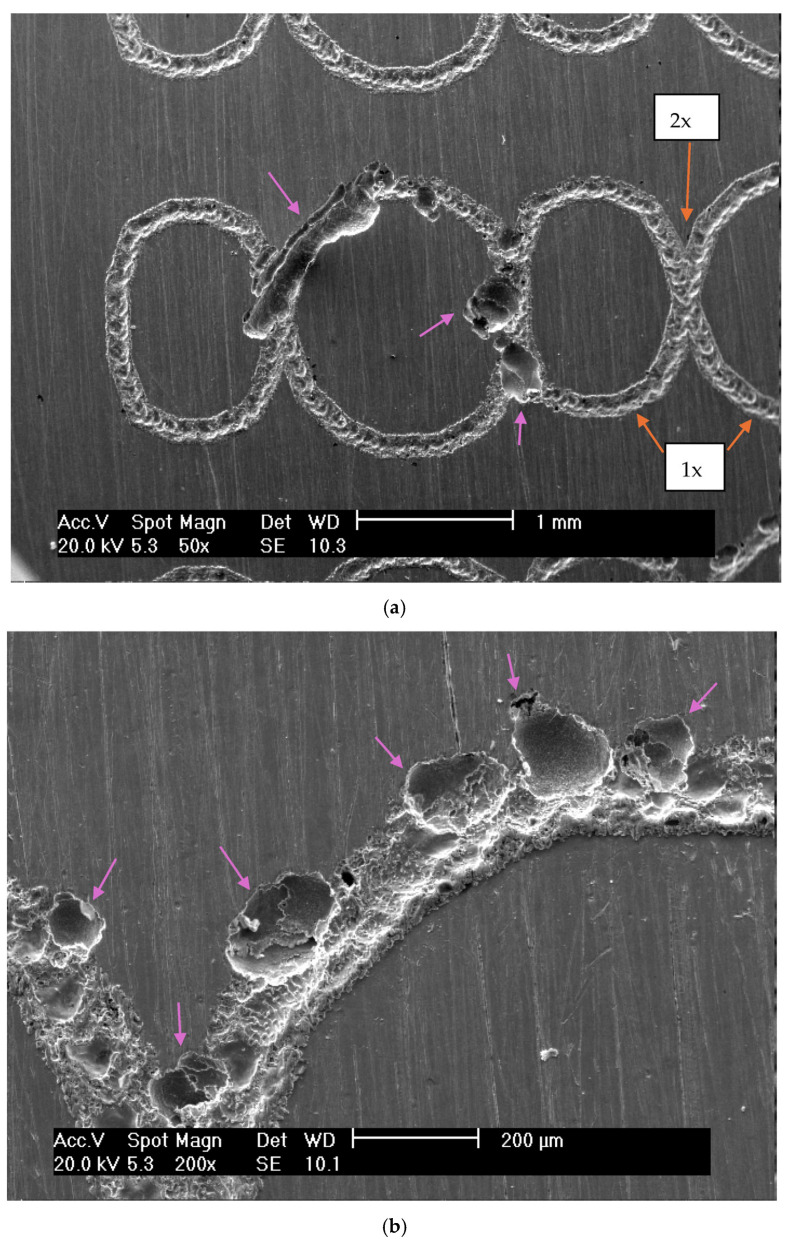

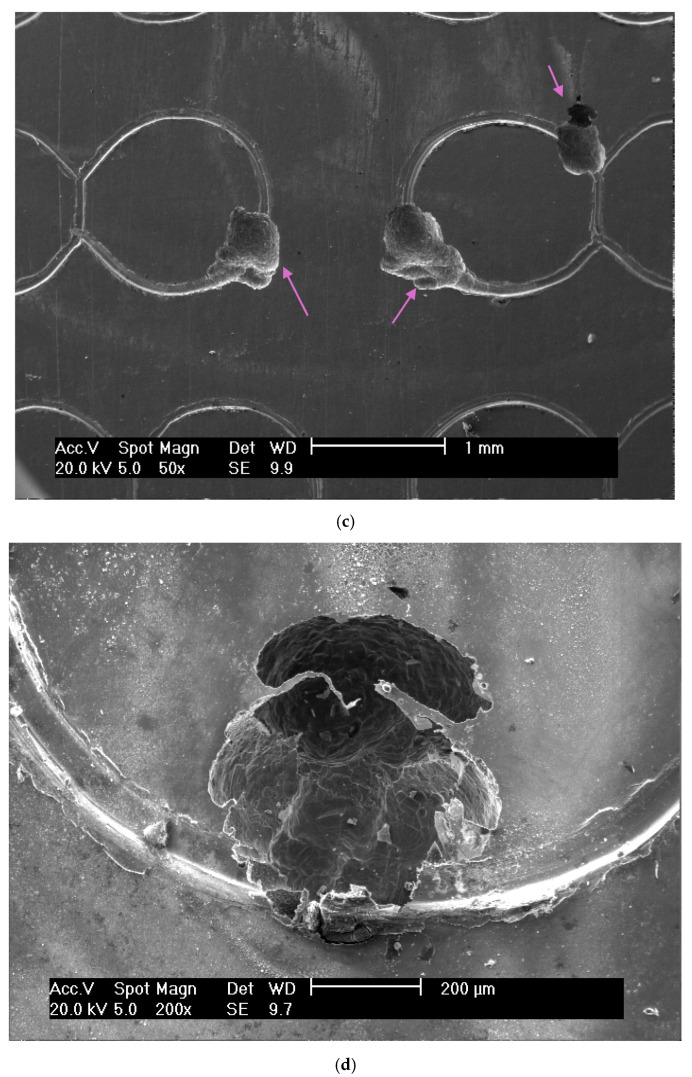

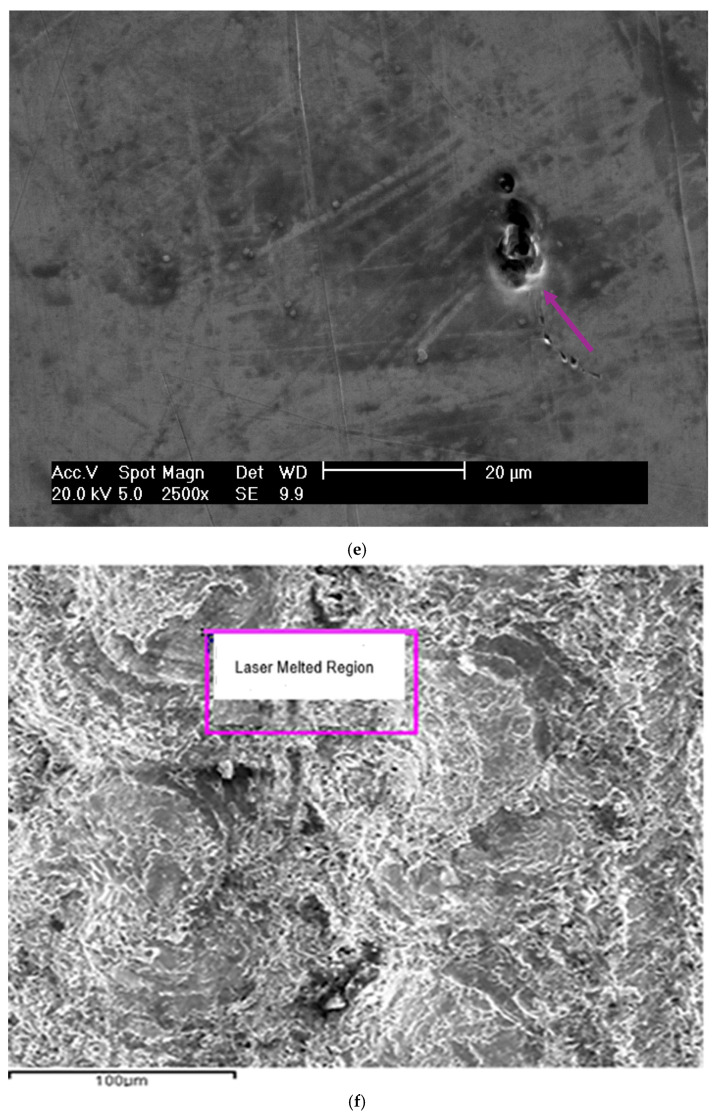

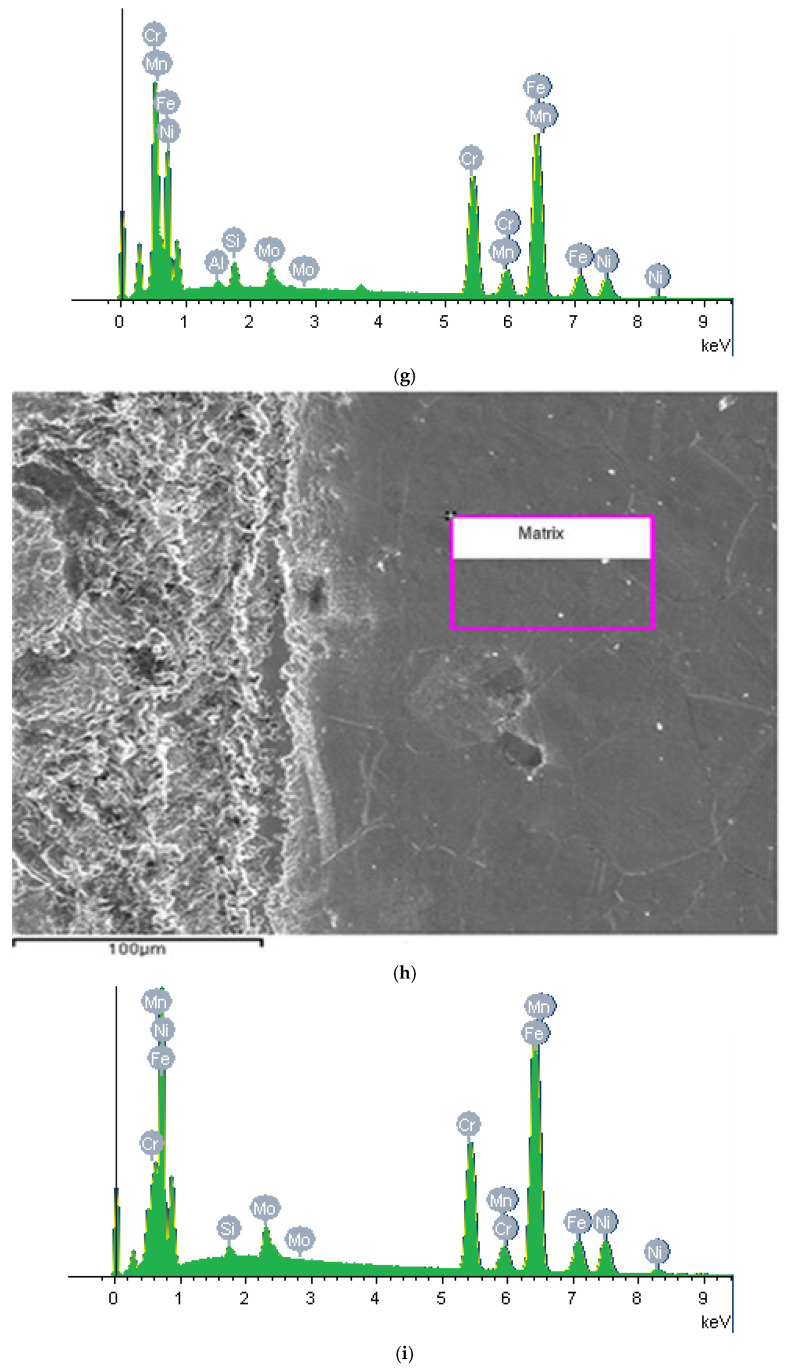
Micrograph images of the ISO 5832-1 stainless steel marked by a laser beam, mechanically and without marks (blank) after electrochemical tests in PBS at 37 °C and semi-quantitative analysis of the EDX spectrum prior to electrochemical tests. (**a**) Corrosion pits formed on laser-related regions for the laser-marked specimen after the cyclic polarization test. The arrows also present the regions where the laser beam passed once or twice. (**b**) Corrosion pits generated on the laser-marked specimen after the cyclic polarization test. (**c**) Corrosion pits formed on a mechanically marked specimen after the cyclic polarization test. (**d**) Corrosion pits generated on a mechanically marked specimen after the cyclic polarization test. (**e**) Corrosion pits formed on a non-marked (blank) specimen after the cyclic polarization test. (**f**) Magnification of the laser-marked region (without electrochemical tests). (**g**) EDX spectrum for the selected region presented in (**f**). (**h**) Blank region. (**i**) EDX spectrum for the selected region presented in (**h**).

The susceptibility of the marked specimens to pitting was evidenced by the observed current oscillations preceding the breakdown of the protective layer. These results indicated that the passive film formed on laser-marked specimens had more defects than those on the mechanical and blank specimens, as Figure 8 shows. A lower pitting resistance is linked to the laser-marked samples. This behaviour was confirmed by the XPS data, as shown in Table 4.

The development and characteristics of the chromium oxide-based passive film are determined by the oxidation process, which is intrinsically connected to the chemical composition of the oxide passive layer.

Once the electrical potential surpasses a critical threshold, chloride ions migrate toward the interface between the metal and passive films, causing the formation of a metal chloride phase. This phase fractures the overlying film due to its large specific volume. Consequently, the chloride phase serves as an accessible source of chloride ions, which facilitate both the initiation and stabilization of pit growth. Following pitting nucleation, these pits expand to form microscale cavities and later undergo repassivation, resulting in stable, growing pits [44]. The presence of metastable pitting is evident in both mechanically marked and laser-marked samples.

When the equilibrium between the breakdown and repair of passive films at defect sites is disturbed, stable pits may develop. Within these pits, locally activated regions function as the anode, whereas the remainder of the passivated surface acts as the cathode. This establishes an electrochemical corrosion cell characterized by a relatively large cathodic area and a small anodic area inside and outside the pit. As anodic reactions progress, metal cations such as Fe^2+^ accumulate within the pit, attracting chloride ions to preserve charge neutrality. The hydrolysis of these cations, coupled with the lack of localized cathodic reactions, results in a decreased pH within the pit [44]. This acidic, chloride-enriched microenvironment further promotes pit propagation through an autocatalytic process.

The EDX spectrum displays data obtained from the selected region of Figure 8f. This technique has identified Al and Si concentrations, which are present as ceramic inclusions of Al_2_O_3_ and SiO_2_. These ceramic inclusions can be easily detached during wear tests or serve as potential sites for initiating corrosive processes. In non-marked regions, Al concentrations were not detected, as shown in Figure 8i. The small Al and Si content was expected, since it aligns with the standards [43]. EDX data also indicated the enrichment of Cr and Mn and the depletion of Mo and Fe in the laser-marked area.

**Figure 9:**
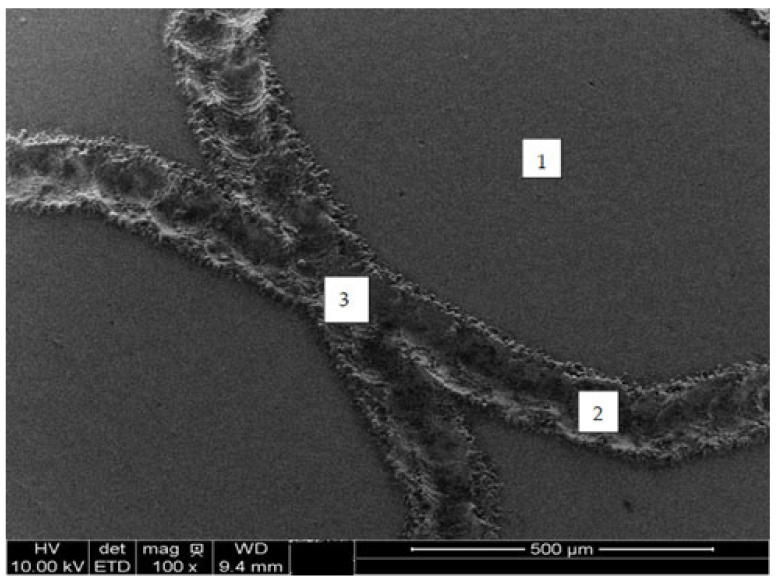
Laser-marked region selected for XPS analyses, prior to electrochemical tests, without etching.

The laser marking process resulted in observable changes in the chemical composition of the passive film, as indicated in Table 4. These data, obtained by XPS from various regions of the samples before cyclic polarization, are depicted in Figure 9. Three areas were examined, namely (1) the matrix, i.e., pristine specimens, (2) laser-marked regions, and (3) laser-marked regions with double laser incidence.

Analyzing Cr/Fe ratio variations across selected surface regions reveals differences in oxide layers. The Cr/Fe ratio increases with the incidence of the laser beam. Similar surface composition atomic percentages were found by Swayne et al. [44] for samples of AISI 316L treated with a Q-switched Nd-YAG laser.

When a laser beam interacts with stainless steel, it can cause localized heating and oxidation of the surface. Chromium, being more reactive than iron at elevated temperatures, tends to preferentially oxidize, leading to an enrichment of Cr_2_O_3_ in the oxide layer. This enrichment can result in a higher Cr/Fe ratio at the surface, especially at the centre of the laser spot where temperatures are highest.

An increase in the Cr/Fe ratio in the laser area of stainless steel typically indicates a change in the surface composition, often due to laser-induced oxidation. This change can significantly impact the material’s corrosion resistance, with higher Cr/Fe ratios often correlating with improved corrosion protection and lower Cr/Fe in the surrounding areas, creating micro-cells where pits can nucleate and propagate [45,46,47].

Suslov et al. [48] observed that higher laser energy density leads to a decrease in Cr and Fe metallic phases in the subsurface layer. This phenomenon results from enhanced oxide formation during laser-induced thermal exposure in an oxygen-rich environment. An increase in energy density results in a reduction in chromium oxide content. This can be attributed to the different oxidation sensitivities of iron and chromium, with iron requiring higher temperatures to oxidize. It is important to note that the discrepancies between our findings and those of other researchers arise from their inadequate specification of the exact locations chosen for analysis on the laser-treated surfaces. In this study, these regions are clearly outlined, as shown in Figure 8 and Figure 9.

This study aimed to assess the impact of two marking techniques on the wear and electrochemical resistance of ISO 5832-1 stainless steel. It was observed that laser markings decrease resistance to pitting corrosion by significantly altering the passive film’s properties compared to unmarked material.

Pulsed laser marking processes are fast and extremely localized. Some chemical elements, like Cr, Mo, and Mn, concentrate in an area and impoverish the surrounding areas. Thus, the electrochemical reactions occurred at regions near the laser-marked sites. Furthermore, the pits are more prone to nucleate and propagate in the areas where the laser has passed twice.

Oxide inclusions became prominent due to the dual incidence of the laser beam. Attacking the steel matrix around inclusions forms micro-crevices that promote pitting growth and propagation. The roughness of the surface potentiates the electrochemical attack. This was the mechanism proposed to explain the increased susceptibility to localized corrosion at the laser-affected areas.

Wear and corrosion constitute additional phenomena arising from the interaction between metallic biomaterials and biological tissues [49].

## 4. Conclusions

The tribological behaviour of this biomaterial depends on the marking process used, with the wear rate affected by both the normal force and the type of sphere employed. Surface characterization revealed changes in the microstructure caused by the high temperatures from laser marking. Although none of the samples were considered cytotoxic, the laser-marked surfaces showed the lowest cellular viability among all tested samples.

The laser engraving technique causes significant changes in the surface’s chemical composition, leading to a high density of defects and increased roughness. The surface inclusions are mainly oxides. These ceramic inclusions are not melted by the rapid laser beam and remain visible on the surface. Alterations in the material’s microstructure reduce its resistance to pitting corrosion, causing the passive film to break down. Under open-circuit potential (OCP) conditions, the marked surfaces act as anodic sites, actively dissolving in a phosphate-buffered saline (PBS) medium.

## Figures and Tables

**Table 1:** Hardness of the materials used in this study.

	Material	Hardness
Sample	ISO 5832-1 SS	88 HRB
Ball	AISI 316L SS	25–39 HRC
Ball	Polypropylene	R85

**Table 2:** Selected test conditions for the ball-cratering wear experiments.

Ball-Cratering Parameters	
(N) Normal Force [N]—Ball of AISI 316L SS	0.40
(N) Normal force [N]—ball of PP	0.05
(S) Sliding distance [m]	60.00
(n) Ball rotational speed—[rpm]	75.00
(v) Tangential sliding speed—[m/s]	0.10
(t) Test duration—[min]	10

**Table 3:** Values of pH for the extracts at a concentration of 100%.

Extracts	pH
CP (Phenol 0.5%)	7.50
CN (Pure Ti)	7.50
Mechanically marked	7.88
Laser-marked	7.88

**Table 4:** Atomic percentage of chemical elements presented in ISO 5832-1 stainless-steel oxide layer measured by XPS.

Element (Atomic%)/Selected Region	1	2	3
C	9.67	3.75	3.99
Cr	13.68	16.87	21.62
Fe	14.86	14.31	11.28
Mn	0.62	1.32	2.07
Mo	1.23	0.27	0.34
N	3.38	3.51	3.39
Ni	4.06	5.56	5.88
O	50.29	51.00	47.80
P	2.21	3.41	3.63
Cr/Fe	0.92	1.18	1.92

## Data Availability

The original contributions presented in the study are included in the article, further inquiries can be directed to the corresponding author.
